# Individuals with Metabolically Healthy Overweight/Obesity Have Higher Fat Utilization than Metabolically Unhealthy Individuals

**DOI:** 10.3390/nu8010002

**Published:** 2016-01-04

**Authors:** Arturo Pujia, Carmine Gazzaruso, Yvelise Ferro, Elisa Mazza, Samantha Maurotti, Cristina Russo, Veronica Lazzaro, Stefano Romeo, Tiziana Montalcini

**Affiliations:** 1 Department of Medical and Surgical Science, University Magna Grecia, Catanzaro 88100, Italy; pujia@unicz.it (A.P.); yferro@unicz.it (Y.F.); elisamazza@inwind.it (E.M.); samabiotec@yahoo.it (S.M.); cristina_russo_cr@libero.it (C.R.); romeo@unicz.it (S.R.); 2 Clinical Institute “Beato Matteo”, Vigevano 27029, Italy; c.gazzaruso@gmail.com; 3 Department of Health Science, University Magna Grecia, Catanzaro 88100, Italy; veronicalazzaro09@gmail.com; 4 Department of Molecular and Clinical Medicine, University of Gothenburg, Gothenburg 40530, Sweden

**Keywords:** obesity, nutrition assessment, fat utilization, Metabolic Syndrome, metabolically unhealthy Obesity, diabetes

## Abstract

The mechanisms underlying the change in phenotype from metabolically healthy to metabolically unhealthy obesity are still unclear. The aim of this study is to investigate whether a difference in fasting fat utilization exists between overweight/obese individuals with a favorable cardiovascular risk profile and those with Metabolic Syndrome and Type 2 diabetes. Furthermore, we sought to explore whether there is an association between fasting fat utilization and insulin resistance. In this cross-sectional study, 172 overweight/obese individuals underwent a nutritional assessment. Those with fasting glucose ≥126 mg/dL or antidiabetic treatment were considered to be diabetics. If at least three of the NCEP criteria were present, they had Metabolic Syndrome, while those with less criteria were considered to be healthy overweight/obese. An indirect calorimetry was performed to estimate Respiratory Quotient, an index of nutrient utilization. A lower Respiratory Quotient (*i.e.*, higher fat utilization) was found in healthy overweight/obese individuals than in those with Metabolic Syndrome and Type 2 diabetes (0.85 ± 0.05; 0.87 ± 0.06; 0.88 ± 0.05 respectively, *p* = 0.04). The univariate and multivariable analysis showed a positive association between the Respiratory Quotient and HOMA-IR (slope in statistic (B) = 0.004; β = 0.42; *p* = 0.005; 95% Confidence interval = 0.001–0.006). In this study, we find, for the first time, that the fasting Respiratory Quotient is significantly lower (fat utilization is higher) in individuals who are metabolically healthy overweight/obese than in those with metabolically unhealthy obesity. In addition, we demonstrated the association between fat utilization and HOMA-IR, an insulin resistance index.

## 1. Introduction

Epidemiological research established that overweight and obese individuals do not always show high rates of cardiovascular diseases (CVD) and mortality [1,2]. Those without dyslipidemia, insulin resistance, and hypertension are characterized by a low risk, despite the presence of an elevated body mass index (BMI) [3]. However, this phenotype seems to be a transient state [4,5] since a high risk of developing Type 2 diabetes mellitus (T2DM) has been demonstrated in those who maintain an unhealthy lifestyle over time [5]. The mechanism underlying the switch in phenotype from the metabolically healthy status to T2DM is still unclear. Obesity, inflammation, and worsening of insulin resistance are recognized as important risk factor in the pathogenesis of diabetes [6,7] but other mechanisms could play a role. A high fat (HF) diet results in an increase in β-oxidation [8]. However, other investigations demonstrated a reduction in β-oxidation [9,10,11,12,13,14,15,16]. These studies are not conflicting because the mechanisms described above could be sequential (first it increases and then, it decreases), leading to the switch from a metabolically healthy but overweight/obesity status to T2DM. In this regard, it is well known that nutrient utilization can be assessed with Indirect Calorimetry by measuring the ratio between carbon dioxide production and oxygen consumption (Respiratory Quotient (RQ)) [17]. Some investigations have demonstrated that subjects who tend to burn less fat have an increased RQ value [18,19]. High RQ is associated with a high rate of subsequent weight gain [20]. Recently, a high post-absorptive RQ was associated with hypertension [21] and increased Carotid Intima-Media Thickness (CIMT), a well-known predictor of cardiovascular events [22,23] in individuals with obesity [24]. Furthermore, fasting RQ is higher in individuals with obesity and hypertriglyceridemia [25] and in overweight/obese individuals with cardiac remodelling than in those who are just obese [26]. In this study, we sought to investigate whether a difference in RQ (and thus, in fat utilization) exists between overweight/obese individuals with a favorable cardiovascular risk profile and those with Metabolic Syndrome (MS) and T2DM, and whether RQ is associated with insulin resistance. This investigation could be useful to hypothesize the mechanisms underlying the progression from a metabolically healthy but overweight/obese phenotype towards metabolically unhealthy obesity and T2DM, and probably to distinguish subjects who will be at a high risk for T2DM and cardiovascular diseases.

## 2. Methods

In this cross-sectional study, the population consisted of white overweight/obese subjects who were undergoing health-screening tests at our outpatient nutrition clinic. All the participants were over 45 years old with a BMI of more than 24.9. Participants underwent a medical interview and the nutritional assessment to verify if there had been any changes in their food habits or if they followed a special diet or used any dietary supplements in the three months prior to our tests. We enrolled consecutively only those who had not performed these actions. All enrolled individuals had the same diet, determined by nutritional intake assessment, *i.e.*, a solid-food diet that supplied 50%–55% of the calories as carbohydrate, 18%–20% as protein, and no more than 30% as fat.

All patients included in the study were not suffering from any diseases (like chronic obstructive pulmonary disease, thyroid dysfunction, cancer, congestive heart failure, myocardial infarction, stroke) and did not take any drug (anti-obesity medications, psychotropic drugs and chronotropic agents)which could affect respiratory gas exchange or had debilitating diseases known to affect blood pressure or plasma glucose or lipid concentrations (like stage 2–5 chronic kidney disease and end stage liver failure) as determined by medical history, a physical examination, and laboratory tests.

Furthermore, we assessed the presence of the known classical cardiovascular (CV) risk factors, MS presence and anthropometric characteristics. The following criteria were used to define the distinct CV risk factors: diabetes: fasting blood glucose ≥126 mg/dL or antidiabetic treatment; hyperlipidemia: total cholesterol >200 mg/dL and/or triglycerides >200 mg/dL or lipid lowering drugs use; hypertension: systolic blood pressure ≥130 mmHg and/or diastolic blood pressure ≥85 mmHg or antihypertensive treatment; overweight: 25 kg/m^2^ ≤ BMI < 30 kg/m^2^; obesity: body mass index (BMI) ≥30 kg/m^2^; smoking: a current smoker who has smoked more than 100 cigarettes in their lifetime and smokes cigarettes every day or some days [27,28].

The selection criteria for MS individuals were based on the National Cholesterol Education Program’s (NCEP) Adult Treatment Panel III report (ATP III). Individuals with 0–2 cardiometabolic abnormalities were identified as having a metabolically healthy but overweight/obese phenotype, while those with at least three or more abnormalies were identified as having MS [29]. Furthermore, all participants underwent the instrumental evaluation of the carotid intima-media thickness (CIMT).

Therefore, in this study we enrolled 172 overweight/obese subjects, categorized into the following three groups: healthy overweight/obese (with maximum two NCEP abnormalities and without T2DM); MS (with three or more NCEP abnormality and without T2DM) and T2DM (only those with fasting glucose ≥126 mg/dL or antidiabetic treatment). Written informed consent was obtained. The protocol was approved by local ethic committee at the University Hospital (projects codes 2013-1/CE). The investigation conforms to the principles outlined in the Declaration of Helsinki.

### 2.1. Blood Pressure Measurement

The measurement of the systemic blood pressure (systolic blood pressure (SBP) and diastolic blood pressure (DBP)) of both arms was obtained by an auscultatory blood pressure technique with aneroid sphygmomanometer. Clinic BP was obtained in supine patients, after 5 min of quiet rest. A minimum of three BP readings were taken using an appropriate BP cuff size (the inflatable part of the BP cuff covered about 80 percent of the circumference of upper arm) as previously described [30].

### 2.2. Biochemical Evaluation

Venous blood was collected after fasting overnight into vacutainer tubes (Becton & Dickinson, Plymouth, UK) and centrifuged within 4 h. Serum glucose, total cholesterol, high density lipoprotein (HDL)-cholesterol, and triglycerides were measured with enzymatic colorimetric test. Low-density lipoprotein (LDL) cholesterol level was calculated by the Friedewald formula: total cholesterol—HDL cholesterol—(triglycerides/5). Plasma insulin concentration was determined by radioimmunoassay. We calculated Homeostasis Model Assessment of Insulin Resistance (HOMA-IR) by the following formula:

HOMA-IR = Fasting blood glucose (mg/dL) × insulin (U/mL)/405

Quality control was assessed daily for all determinations.

### 2.3. Anthropometric Measurements

All tests were performed after a 12 h overnight fast. Body weight was measured with a calibrated scale with the subjects lightly dressed, subtracting the weight of clothes. Height was measured with a wall-mounted stadiometer (TANITA, Middlesex, UK). BMI was calculated with the following formula: weight (kg)/height (m)^2^. Waist circumferences and hip circumferences (WC and HC) were measured with a nonstretchable tape over the unclothed abdomen at the narrowest point between costal margin and iliac crest at the level of the widest diameter around the buttocks, respectively [31]. Bioelectrical impedance analysis (BIA) (BIA-101, Akernsrl, Florence, Italy) was performed to estimate the percentage of Total Body Water (TBW), Fat Mass (FM), Muscle Mass (MM), total Fat-Free Mass (FFM) [32].

### 2.4. Dietary Intake Assessment

The participant’s nutritional intake was calculated using nutritional software MetaDieta 3.0.1 (Metedasrl, San Benedetto del Tronto, Italy). Dietary intake data comprised a 24-h recall and a seven-day diet record. The 24-h recall was collected via an interview by a dietitian who used images associated with a comprehensive food list in the program. All participants were also given a food diary, measuring sheet with life-size images of a spoon, cup and bottle sizes for food diaries. The INRAN (National Institute of Food Research) 2000 and IEO (European Institute of Oncology) 2008 database serves as the source of food composition information in the program. The data was entered by dietitians into the program. All foods are assigned a unique code which allows categorization of foods into food groups. The resulting database was exported into SPSS (IBM Corporation, New York, NY, USA) for analysis.

### 2.5. RQ Assessment—Indirect Calorimetry

RQ and the Resting Energy Expenditure (REE) were measured by Indirect Calorimetry using the open circuit technique (Viasys Healthcare, Hoechberg, Germany). All tests were performed after fasting overnigh, between hours of 7 a.m. and 8:30 a.m. after 48 h abstention from exercise, in a sedentary position. The participant rested quietly for 30 min in an isolated room at a controlled temperature (21–24 °C). Respiratory gas exchange was measured within a canopy circuit for at least 30 min, until steady state was achieved. The calorimeter quantifies the volume of O2 inspired and CO2 expired by the subject. Resting Energy Expenditure is calculated by the Weir formula. RQ was calculated as CO2 production/O2 consumption. Criteria for a valid measurement was at least 15 min of steady state, with less than 10% fluctuation in minute ventilation and oxygen consumption and less than 5% fluctuation in RQ [26,33].

### 2.6. Carotid Arteries Assessment

The participants underwent B-mode ultrasonography of the extracranial carotid arteries by use of a high-resolution ultrasound instrument (Toshiba Medical Systems Corporation, model TUS-A500, 1385, Shimoishigami, Otawara-Shi, Tochigi, Japan). We used a 5- to 12-MHz linear array multifrequency transducer. All the examinations were performed by the same ultrasonographer blinded to clinical information with patients in the supine position. ECG leads were attached to the ultrasound recorder for on-line continuous heart rate monitoring. The right and left common (CCA) and internal carotid arteries (including bifurcations) were evaluated with the head of the subjects turned away from the sonographer and the neck extended with mild rotation. The IMT, defined as the distance between intimal-luminal interface and medial—adventitial interface, was measured as previously described [24]. In posterior approach and with the sound beam set perpendicular to the arterial surface, 1 cm from the bifurcation, three longitudinal measurements of IMT were completed on the right and left common carotid arteries far-wall, at sites free of any discrete plaques. The mean of the three right and left longitudinal measurements was then calculated. Then, we calculated and used for statistical analysis the mean CIMT between right and left CCA. The coefficient variation of the methods was 3.3%. 

## 3. Statistical Analysis

Data is reported as mean ± SD. Thirty subjects for each group are required to detect a significant difference of RQ greater than 2% (21–26) with 80% power on a two-sided level of significance of 0.05. A chi-square test was performed to analyze the prevalence of the cardiovascular risk factors and medications. ANOVA was performed to compare the means between groups with a Fisher’s LSD test as a post-hoc analysis. REE and RQ values were eventually adjusted according to the difference in FFM between groups or if RQ and REE correlated with FFM.

The Pearson’s correlation was used to identify the variables correlated with RQ given that the continuous variables were normally distributed. We analyzed the correlation with the following variables: REE, FFM, age, BMI, WC, glucose, LDL, HDL, triglycerides, PAS, PAD, HOMA-IR. Stepwise multivariable linear regression analysis was used to test the association between RQ and the variables selected among those correlated with RQ in the univariate analysis, with *p* < 0.1. When we tested the association with HOMA-IR and cardiometabolic risk factors, glucose was excluded since it was considered as part of HOMA-IR. Significant differences were assumed to be present at *p* < 0.05 (two-tailed). All comparisons were performed using SPSS 20.0 for Windows (IBM Corporation, New York, NY, USA).

## 4. Results

Among the participants, we enrolled 80, 58, and 34 individuals who were overweight/obese, with MS and Type 2 Diabetes, respectively. Since we did not find any difference of RQ between gender and between individuals taking medications or not (data not shown) we presented the data altogether.

The demographic and anthropometric characteristics, the prevalence of cardiovascular risk factors, and medications use of the population are indicated in Table 1. Healthy overweight/obese had a lower RQ than those with MS and Type 2 diabetes (*p* = 0.04; ANOVA, Table 2). In particular, healthy overweight/obese had a lower RQ than MS (*p* = 0.04; post-hoc analysis) and a lower RQ than T2DM (*p* = 0.03; *post-hoc* analysis; Table 2), respectively. FFM did not differ between groups (*p* = 0.92). Furthermore, RQ and FFM (as absolute value) did not correlate (*r* = 0.11 and *p* = 0.27). As expected, CIMT were significantly higher in T2DM than in MS (*p* = 0.03; post-hoc analysis) and the healthy overweight/obese (*p* = 0.02; post-hoc analysis).

Table 3 shows the factors significantly associated with RQ in the univariate analysis, which were the following: HOMA-IR, glucose, triglycerides, SBP. In the multivariable analysis, RQ remained still associated with HOMA-IR, while triglycerides and SBP were not associated (Table 4).

## 5. Discussion

In this investigation, we find that fasting RQ, an index of nutrient utilization assessed by indirect calorimetry, is significantly lower in individuals with metabolically healthy overweight/obesity than in those with MS and T2DM. This suggests that individuals who are healthy overweight/obese are still able, to some extent, to utilize fat in the fasting state while fat utilization is significantly reduced in individuals with unhealthy obesity (Table 2). These results could help to hypothesize that new factors are involved in the pathogenesis of T2DM and potential new therapeutic goals exist. Furthermore, in this population, we demonstrated the association between RQ and HOMA-IR, which is widely utilized as an insulin resistance index (Table 4). This result could have important implications in predicting diabetes, which must be confirmed by longitudinal studies. The mechanisms underlying the switch in phenotype from healthy overweight/obese to T2DM are still unknown and our study was not designed to investigate these mechanisms. However, our study may be useful in generating intriguing hypotheses. Whether [34,35] or not [36,37,38,39] increase in fatty acid β-oxidation leads to insulin resistance is still a subject of debate. There is evidence that obesity-associated glucose intolerance might develop from an overload of fatty acid in muscle mitochondria [40]. It has been demonstrated that the excessive availability of fatty acids may exert an insulin-desensitizing action in muscle mitochondria [8]. Furthermore, it has been demonstrated that a HF diet and/or obesity can increase the expression of several β-oxidative enzymes [41] and reduce RQ [8]. It is interesting that these events precede the onset of insulin resistance [8]. Our findings are in line with these studies since we find that individuals who are metabolically healthy overweight/obese have, to some extent, a greater ability to burn fat (lower RQ) in comparison to those with MS and T2DM. However, it has also been demonstrated that an unhealthy lifestyle, including HF feeding and the absence of physical activity, favors incomplete β-oxidation caused by the mismatch between β-oxidation and tricarboxylic acid cycle activity, contributing to mitochondrial damage [41,42]. Incomplete fatty acid oxidation also facilitates the production of reactive oxygen species (ROS) which can cause damage to mitochondrial enzymes [42]. Furthermore, the production of ROS represents a common pathway in the cascade of events that finally results in β-cell failure [43]. Consequently, as confirmed by other investigations, both glucose-tolerance and fat oxidation are decreased [9,10,11,44,45,46]. Together these studies lead us to hypothesize that a reduction in fatty acid oxidation is achieved over time, probably in the context of an unhealthy lifestyle. The significant difference of RQ (of fasting fat utilization) between metabolically healthy but overweight/obese phenotype, with MS and T2DM individuals may confirm this mechanism.

**Table 1:** Demographic, anthropometric and clinical characteristics of the population.

Variables	Overweight/Obese (OO) (*n* = 80)	Metabolic Syndrome (MS) (*n* = 58)	T2 Diabetes (T2DM) (*n* = 34)	P ANOVA	*p* Post-Hoc Analysis
Females (%)	31.3	34.5	35.3	0.63	/
Age (years)	56 ± 10	58 ± 9	62 ± 10	0.016	OO *vs.* T2DM 0.004
Weight (Kg)	83 ± 17	87 ± 21	85 ± 20	0.505	/
BMI (Kg/m^2^)	33 ± 6	34 ± 7	34 ± 6	0.514	/
WC (cm)	102 ± 14	106 ± 15	108 ± 13	0.120	/
HC (cm)	109 ± 15	110 ± 12	110 ± 11	0.905	/
SBP (mmHg)	122 ± 11	133 ± 15	130 ± 13	<0.001	OO *vs.* MS < 0.001
					OO *vs.* T2DM 0.003
DBP (mmHg)	78 ± 8	80 ± 11	77 ± 9	0.161	/
Glucose-mg/dL (mmol/L)	91 ± 9 (5.06 ± 0.5)	100 ± 10 (5.56 ± 0.5)	130 ± 45 (7.22 ± 2.5)	<0.001	OO *vs.* MS 0.019
					OO *vs.* T2DM < 0.001
					MS *vs.* T2DM < 0.001
Insulin-mU/L (pmol/L)	16 ± 8 (114.7 ± 57)	23 ± 20 (164.9 ± 143)	35 ± 26 (250.9 ± 186)	0.039	OO *vs.* T2D 0.012
HOMA-IR	3.7 ± 2	6 ± 5	12 ± 11	0.004	OO *vs.* T2DM 0.001
					MS *vs.* T2DM 0.017
TotCholesterol-mg/dL (mmol/L)	199 ± 38 (5.14 ± 0.98)	213 ± 42 (5.5 ± 1.09)	195 ± 47 (5.04 ± 1.2)	0.055	OO *vs.* MS 0.042
					MS *vs.* T2DM 0.037
HDL (mmol/L)	1.52 ± 0.41	1.16 ± 0.36	1.37 ± 0.44	<0.001	OO *vs.* MS < 0.001
					OO *vs.* T2DM 0.017
					MS *vs.* T2DM 0.046
LDL (mmol/L)	3.18 ± 0.83	3.39 ± 0.96	2.97 ± 1.06	0.141	/
Triglycerides (mmol/L)	91 ± 28 (1.03 ± 0.32)	201 ± 81 (2.27 ± 0.91)	151 ± 85 (1.70 ± 0.96)	<0.001	OO *vs.* MS < 0.001
					OO *vs.* T2D < 0.001
					MS *vs.* T2D < 0.001
**Prevalence**					
Hypertension (%)	12	26	52	0.001	/
Dyslipidemia (%)	19	29	30	0.129	/
Smokers (%)	38	46	24	0.390	/
Antidiabetic agents (%)	0	0	56	<0.001	/
Antihypertensive agents (%)	0	0	56	<0.001	/
Lipid lowering agents (%)	0	0	44	<0.001	/

Legend: BMI, body mass index; DBP, diastolic blood pressure; HC, hip circumferences; HDL, high density lipoprotein; HOMA IR, Homeostasis Model Assessment of Insulin Resistance; LDL, low density lipoprotein; SBP, systolic blood pressure; Tot Cholesterol, total cholesterol; WC, Waist circumferences.

**Table 2:** Respiratory quotient, resting energy expenditure, body composition, and carotid intima-media thickness according to groups (Overweight/Obese, with Metabolic Syndrome, with Type 2 Diabetes Mellitus).

Variables	Overweight/Obese (OO) (*n* = 80)	Metabolic Syndrome (MS) (*n* = 58)	T2 Diabetes (T2DM) (*n* = 34)	P ANOVA	*p* Post-Hoc Analysis
REE (FFM adjusted; kcal)	1371 ± 33	1392 ± 42	1383 ± 45	0.93	/
RQ	0.85 ± 0.05	0.87 ± 0.06	0.88 ± 0.05	0.042	OO *vs.* MS 0.044
					OO *vs.* T2DM 0.033
TBW (%)	45 ± 10	47 ± 9	47 ± 8	0.596	/
ECW (%)	31 ± 15	32 ± 14	37 ± 14	0.272	/
FFM (%)	59 ± 12	61 ± 12	61 ± 10	0.707	/
MM (%)	38 ± 8	40 ± 8	39 ± 9	0.665	/
FM (%)	36 ± 9	36 ± 8	36 ± 8	0.943	/
FFM (%)	59 ± 12	61 ± 12	61 ± 10	0.707	/
FFM(kg)	50.4 ± 18	52.4 ± 25	51.8 ± 18	0.92	/
CIMT (mm)	0.7 ± 0.2	0.7 ± 0.2	0.8 ± 0.2	0.054	OO *vs.* T2D 0.024
					MS *vs.* T2D 0.038

Legend: CIMT, carotid intima-media thickness; ECW, extracellular water; FFM, free fat mass; FM, fat mass; MM, muscle mass; REE, resting energy expenditure; RQ, respiratory quotient; TBW, total body water.

**Table 3:** Pearson correlation-factors correlated to respiratory quotient.

Variable	Correlation Parameters	Age	REE	BMI	WC	FFM	HOMA-IR	Glucose	LDL	Triglycerides	HDL	SBP	DBP
**RQ**	r	0.07	0.04	−0.03	0.02	0.92	0.42	0.16	−0.03	0.19	−0.11	0.18	0.08
	p	0.35	0.53	0.65	0.79	0.27	0.005	0.03	0.62	0.01	0.13	0.01	0.25

Legend: BMI, body mass index; DBP, diastolic blood pressure; FFM, free fat mass; HDL, high density lipoprotein; HOMA IR, Homeostasis Model Assessment of Insulin Resistance; LDL, low density lipoprotein; REE, resting energy expenditure; RQ, respiratory quotient; SBP, systolic blood pressure; WC, Waist circumference.

**Table 4:** Multivariate linear regression analysis—factors associated with respiratory quotient.

Dependent Variable RQ	B	SE	β	t	p	95% C.I.	
						Lower Limit	Upper Limit
HOMA-IR	0.004	0.001	0.42	2.98	0.005	0.001	0.006
Triglycerides	0.001	0.001	0.20	1.37	0.17	−0.002	0.002
SBP	0.001	0.001	0.05	0.34	0.73	−0.001	0.001

Legend: RQ, respiratory quotients; HOMA-IR, Homeostasis Model Assessment of Insulin Resistance; SBP, systolic blood pressure.

Furthermore, it is well recognized that despite the substantial research efforts in the last 10–15 years, many individuals unavoidably progress to T2DM [47] thus, longitudinal studies are needed to clarify the eventual role of RQ in predicting the risk of diabetes. Additional studies are also needed to find appropriate intervention (dietetic, pharmacological) to maintain the healthy phenotype by increasing fat oxidation [48].

In this study, some strengths and weaknesses must be pointed out. For some researchers, it is important to consider HOMA-IR to define metabolically healthy obese individuals [49]. However, at present there is a lack of consensus on this definition [50,51,52]. According to previous investigations, we used the NCEP ATP III criteria to define individual who were “metabolically healthy” [53,54], taking the CVD risk into account [55]. Our study was limited by cross-sectional design, thus, it is impossible to infer causality. Nevertheless, cross-sectional studies indicate associations that may exist and are therefore useful in generating hypotheses for future research. In addition, in our study the statistical analysis is robust and adequate. Our results were not purely random as established by previous investigations [9,10,11,12,13,14,15,16,20,21,22,23,24,25,26] and were confirmed by multiple statistical analyses. The investigation was carried out on representative samples of the population which originates from a Mediterranean context, potentially increasing knowledge on this issue from a geographical perspective. Finally, our results are in line with those of other authors who demonstrated the association between metabolic inflexibility, which is independently associated with fasting RQ, and insulin resistance [56]. However, to our knowledge, this is the first time that a difference in fasting RQ has been found between individuals who are metabolically healthy but overweight/obese who have MS and T2DM.

## 6. Conclusions

We find that fasting fat utilization is significantly lower in individuals who are metabolically healthy overweight/obese than in those who are metabolically unhealthy. These results can help to hypothesize the factors involved in the pathogenesis of T2DM.
